# Comprehensive anatomical study of meningeal arteries in dromedaries

**DOI:** 10.1038/s41598-023-47145-1

**Published:** 2023-11-13

**Authors:** Ahmad Al Aiyan, Rinsha Balan

**Affiliations:** Department of Veterinary Medicine, College of Agriculture and Veterinary Medicine, United Arab Emirates University, Al Ain, Abu Dhabi, United Arab Emirates

**Keywords:** Neuroscience, Zoology, Anatomy, Neurology

## Abstract

This study provides a detailed, in-depth analysis of the anatomy, topography, and branching patterns of the meningeal arteries in dromedary camels, a subject that has not previously been thoroughly studied in animals, providing insight into the intricate biological adaptations that allow them to survive in harsh environments. By precisely examining 20 heads obtained from freshly slaughtered dromedaries, we revealed the origins and topologies of the rostral, middle, and caudal meningeal arteries using advanced casting techniques for precise rendering. Our findings indicate that the rostral meningeal artery derives from the external ethmoidal artery and primarily supplies the rostrodorsal region of the frontal lobe. The middle meningeal artery provides blood to approximately two-thirds of the brain meninges. The caudal meningeal artery is derived from the occipital artery and supplies the meninges covering the cerebellum, caudal part of the falx cerebri, and tentorium cerebelli. Significantly, our study revealed the presence of accessory branches originating from the rostral epidural rete mirabile, a finding not previously described in the existing literature. These branches supply the meninges of the frontal and lateral regions of the frontal lobes. This novel study advances our understanding of the meningeal arteries in dromedaries and has significant implications for advancements in veterinary neuroscience.

## Introduction

The meningeal arteries play a crucial role in supplying all three layers of the meninges and partially contribute to the blood supply to the central nervous system^1–3^. Recently, there has been an increasing demand for an improved understanding of the anatomy and vascularization of these arteries in various animal species. Camels are exceptional animals with a remarkable adaptability to harsh environmental conditions, exhibiting adaptations that extend specifically to the blood supply to the brain^4–8^. Several studies have focused on the neuroanatomy of this animal. However, the meningeal arteries of camels have received limited attention and a thorough understanding of their distribution and branching patterns is lacking. Investigating the structure and function of meningeal arteries in camels can help us better understand the biological mechanisms that enable them to survive in such extreme conditions.

The meninges are the three membrane layers that cover and protect the central nervous system from trauma by acting as shock absorbers. They stabilize the brain and prevent it from moving around the skull while supporting blood vessels, nerves, and cerebrospinal fluid^1–3^.

Generally, there are three main meningeal arteries: the rostral, middle, and caudal. The rostral meningeal artery supplies the rostral region of the brain, whereas the middle meningeal artery primarily supplies the lateral and basal regions. The caudal meningeal artery provides blood to the caudal brain regions. These arteries originate from different sources and have different entry locations into the cerebral cavity depending on the species and individual anatomical variations. The branches of the meningeal arteries rise between the dura mater and periosteum of the cranial bone, supplying both structures^9^. In addition to supplying blood to adjacent skull structures, meningeal arteries have few anastomoses with the cerebral arteries.

The middle meningeal artery is the largest of these three arteries in humans, and knowledge of its anatomical position and branches is significant in radiology and surgical procedures^10^. The meningeal arteries are particularly sensitive to cranial lesions. The caudal meningeal artery is particularly susceptible to cranial injuries because of its unique anatomical position and proximity to the temporal bone region^11^. Several clinical complications have been associated with meningeal artery involvement, including epidural hematomas^9,12^, aneurysms^13^, and fistulas^9,14^. Understanding the topography of the meningeal artery is essential before performing surgical procedures at the base of the skull. Studying the anatomy of the meningeal arteries in camels can contribute valuable information to the field of comparative anatomy, which is crucial for understanding the evolutionary processes of the central nervous system and its vasculature across different animal species, and holds significant importance for potentially enhancing research related to various neurological conditions in humans^15^.

While no studies have focused on describing the meningeal arteries in animals, some studies have mentioned the role of the middle meningeal artery in forming the rostral mirabile rete structure but did not discuss the branching pattern of this artery^7,16–18^. To the best of our knowledge, this is the first study to provide a detailed description of all three meningeal arteries in dromedaries.

This study aimed to conduct a comprehensive analysis of the anatomy, topography, branching patterns of the meningeal arteries, and anastomoses with other cerebral arteries in dromedaries. By employing advanced casting techniques, we attempted to provide a more accurate and detailed three-dimensional description and elucidate the finer arterial branches of the meningeal arteries that might be overlooked using traditional dissection methods.

## Results

**Figure 1:**
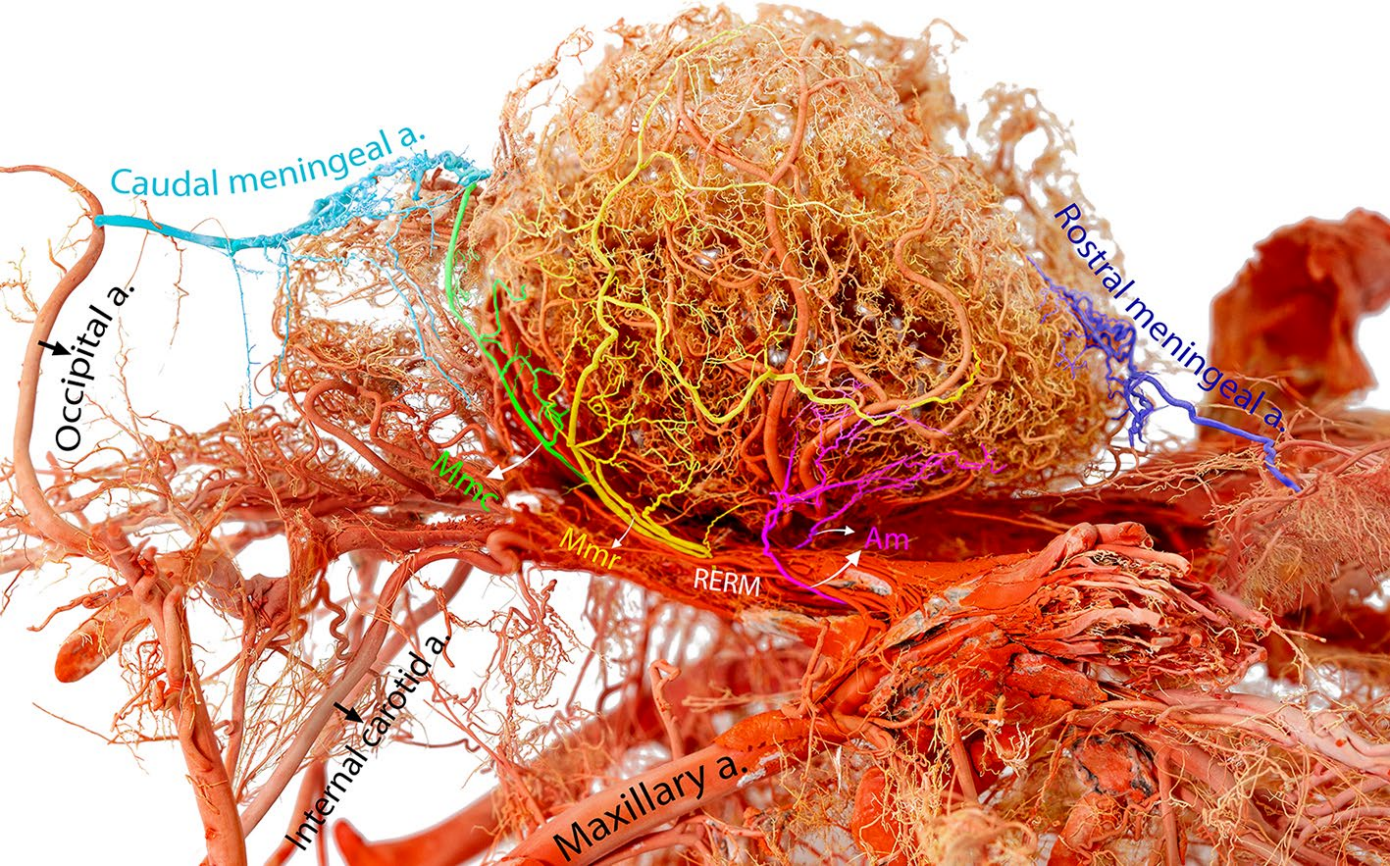
Lateral view of a three-dimensional casting of the dromedary camel brain arterial system. The meningeal arteries are highlighted in different colors to emphasize their unique branching pattern and contribution to the meninges. Mmc, caudal branch of the middle meningeal artery; Mmr, rostral branch of the middle meningeal artery; Am, accessory meningeal arteries; RERM, rostral epidural rete mirabile.

**Figure 2:**
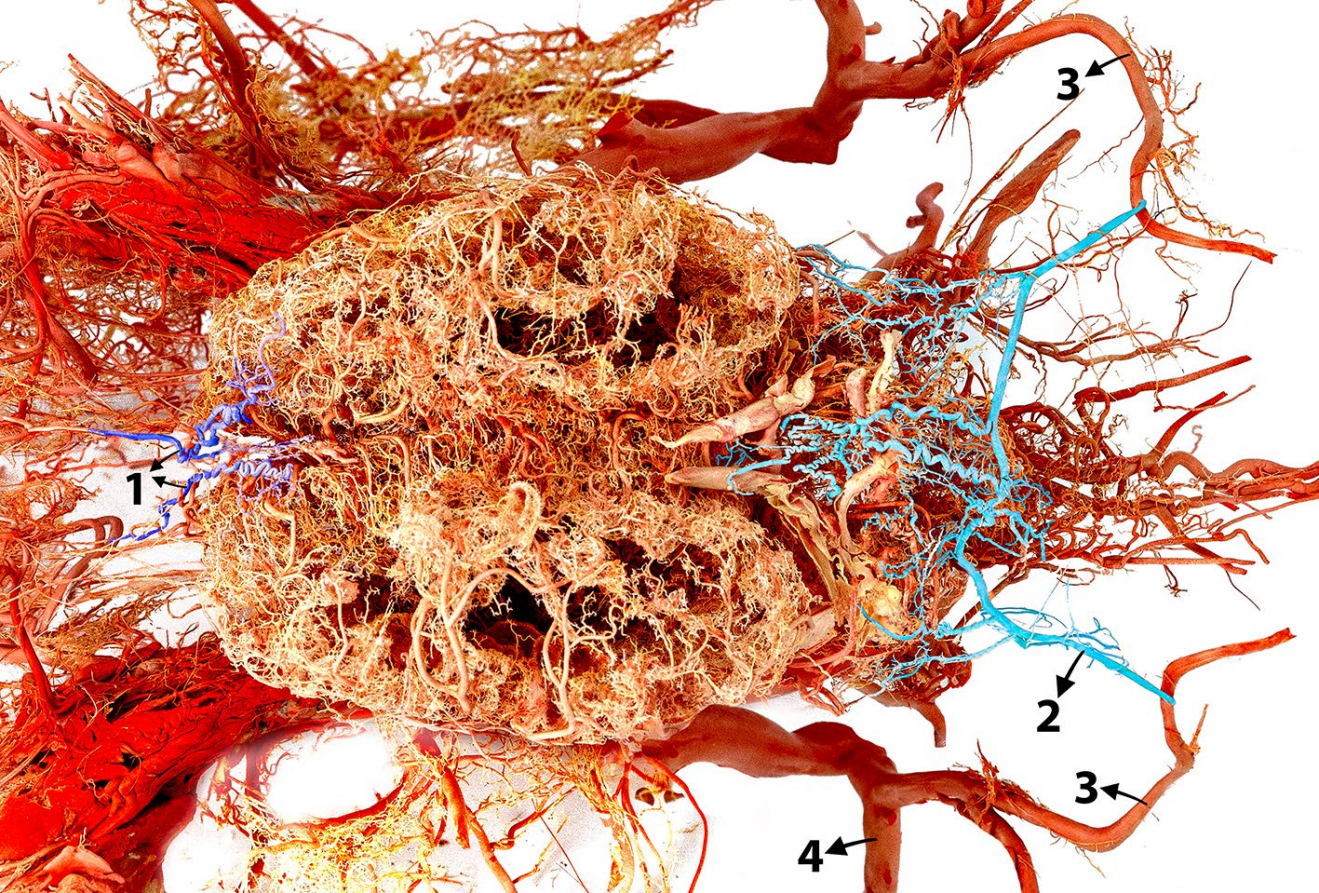
Dorsal view of a three-dimensional casting of the dromedary camel brain arterial system. The rostral and caudal meningeal arteries are highlighted in different colors to emphasize their unique branching patterns and contribution to the meninges. 1, rostral meningeal artery; 2, caudal meningeal artery; 3, occipital arteries; 4, external carotid artery.

**Figure 3:**
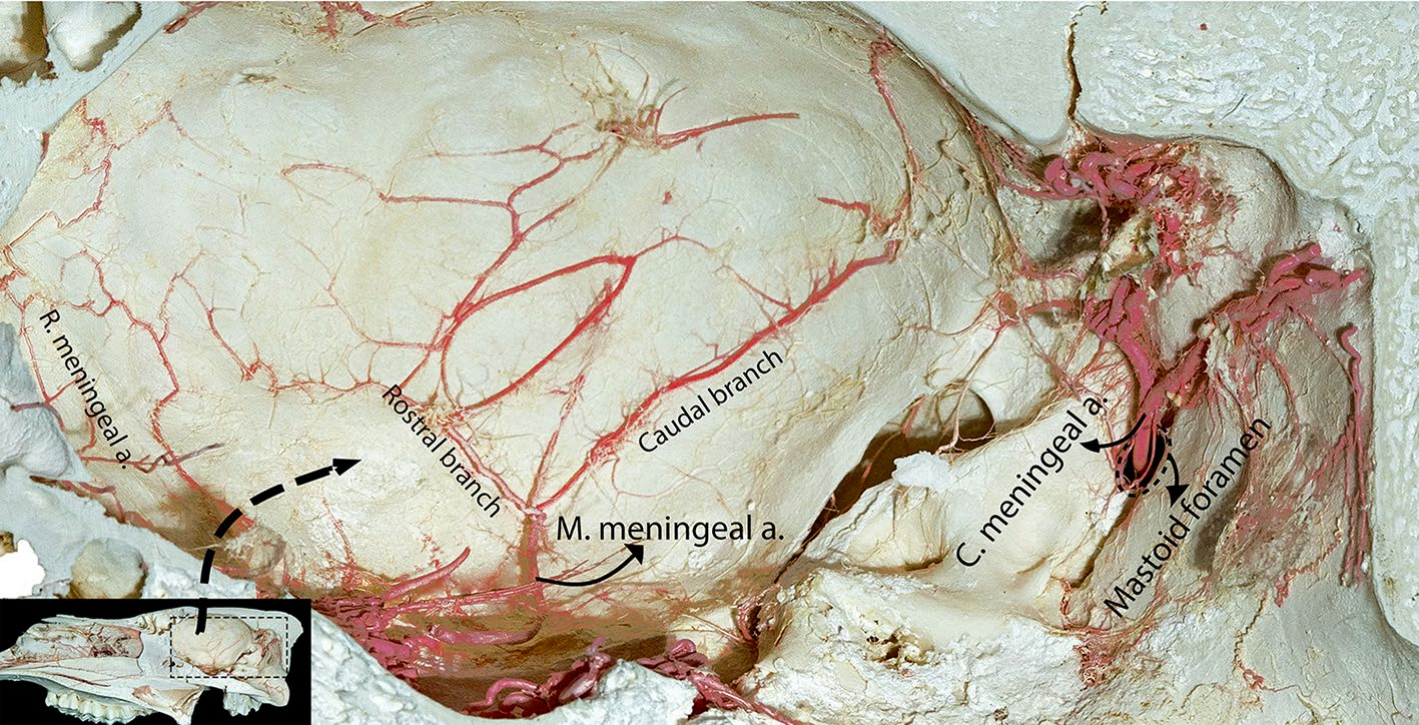
Medial view of the right half of a macerated dromedary camel skull, providing an internal perspective of the casting of the meningeal arteries inside the cranial cavity, allowing for a detailed examination of their spatial distribution and intricate branching pattern.

Our results revealed that the meningeal arteries with diverse origins penetrate the camel skull through distinct foramina, move between the dura mater and bone, and give rise to several branches that supply blood to different regions of the meninges. These arteries are primarily classified into three types: the rostral, middle, and caudal meningeal arteries (Figs. 1, 2, and 3).

**Figure 4:**
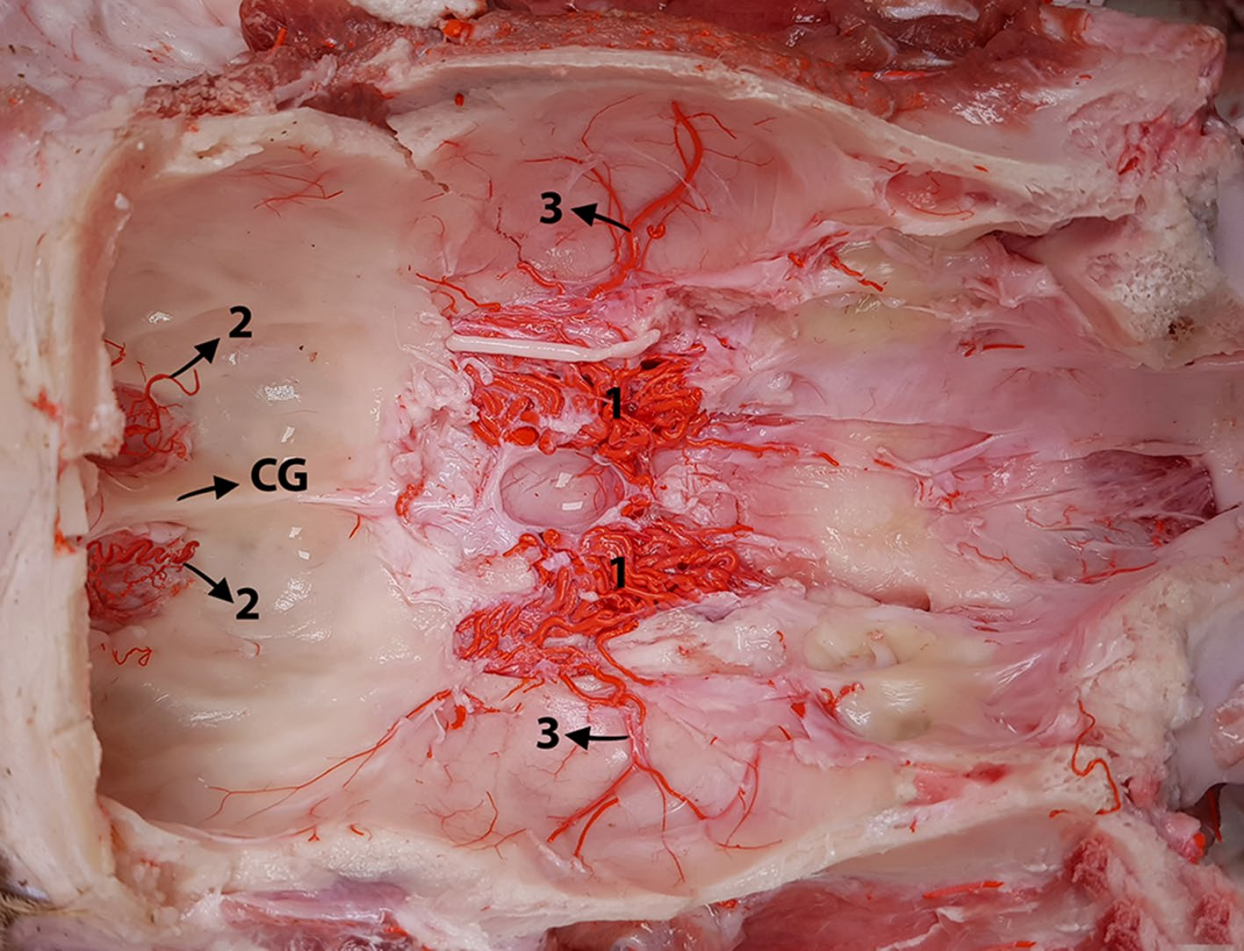
Dorsal view of an exposed cranial cavity of a one-humped camel (with the brain removed), showing the anatomical layout of the rostral and middle meningeal arteries. 1, rostral epidural rete mirabile (RERM); 2, rostral meningeal artery; and 3, middle meningeal arteries; CG, crista galli.

The rostral meningeal artery is derived from the external ethmoidal artery as it passes through the multiple ethmoid foramina (Fig. 4). In turn, the external ethmoidal artery arises dorsally from the maxillary artery and rostral to the emergence of the epidural rete mirabile branches. In our study, we observed that as the external ethmoidal arteries passed through multiple ethmoid foramina, they split into several thin branches. These branches further subdivide and supply the ethmoid bone and the adjacent structures to the ethmoid fossa. Notably, these finer branches curved around, encircled, and supplied the olfactory bulb. Some of these branches join to form the rostral meningeal artery, which climbs toward the cribriform plate. This artery had a tortuous course on both sides, eventually anastomosing with the opposite artery at the crista galli (Fig. 4). Subsequently, the rostral meningeal artery ran posteriorly across the frontoethmoidal region, resulting in a series of twisting branches (Figs. 1 and 2). These branches travel toward the longitudinal fissure of the frontal segments of the cerebral hemispheres, supplying the meninges in the ethmoid fossa and frontal lobe of the cerebrum. A distinct separation was observed between the external and internal ethmoidal arteries, with the latter originating from the rostral cerebral artery.

**Figure 5:**
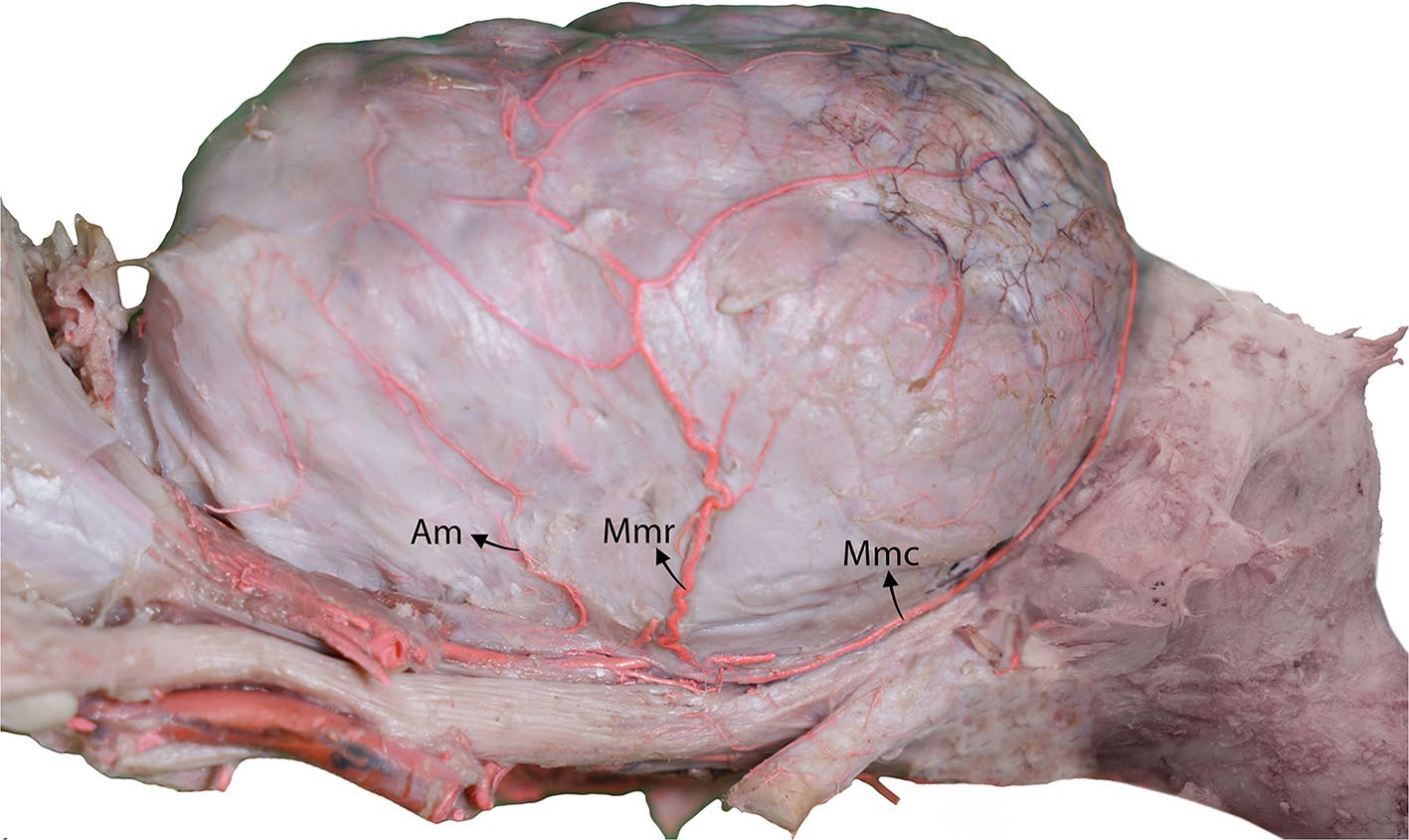
Lateral view of the dromedary camel’s brain showing branching pattern of the middle meningeal artery. Mmc, caudal branch of the middle meningeal artery; Mmr, rostral branch of the middle meningeal artery; Am, accessory meningeal arteries.

We observed variations in the origins of the middle meningeal artery. Our examination revealed that the middle meningeal artery emerged from the rostral epidural rete mirabile (RERM) in most of the studied samples (Fig. 4). The middle meningeal artery was divided into rostral and caudal branches (Figs. 1, 3, and 5). It supplies the meninges, covering the parietal, temporal, and occipital cerebral cortices. In most of the studied cases, we found that the rostral and caudal branches emerged from multiple points of the middle meningeal artery (Fig. 4); in some cases, they arose from a single stem (Fig. 3). In one case, the rostral branch of the middle meningeal artery was derived from the rostral branches of the maxillary artery, which contributed to the formation of the RERM (Fig. 4). In this case, we found that the rostral branch of the middle meningeal artery contributed to the RERM before sending out several branches that supplied the meninges.

Three additional samples revealed the existence of an artery that emerged from the medial side of the maxillary artery, ascended dorsomedially, crossed the lateral region of the tympanic bullae, and entered the cranium through the foramen ovale. In these three samples, the middle meningeal artery entered the cranium, split into several branches, and contributed to the caudal root of the RERM before releasing several branches that supplied the meninges.

The rostral branch had a wider region of supply than the caudal branch in all studied samples (Fig. 5). The rostral branch traversed the middle cranial fossa, specifically the greater wing of the sphenoid bone, and gave rise to its terminal branches, which supplied the meninges that cover the parietal and temporal lobes.

The caudal branch of the middle meningeal artery traveled caudally along the squamous portion of the temporal bone. It extends to the lower margin of the parietal bone, where its terminal branches continue to move backward toward the transverse fissure between the cerebrum and the cerebellum (Fig. 5). These branches provide blood to the meninges in this region. Our findings indicated that, in five of the studied samples, the terminal branches from the caudal branch of the middle meningeal artery were anastomosed with the caudal meningeal artery (Fig. 7). The rostral and caudal terminal branches supplied blood to the cerebellar falx.

**Figure 6:**
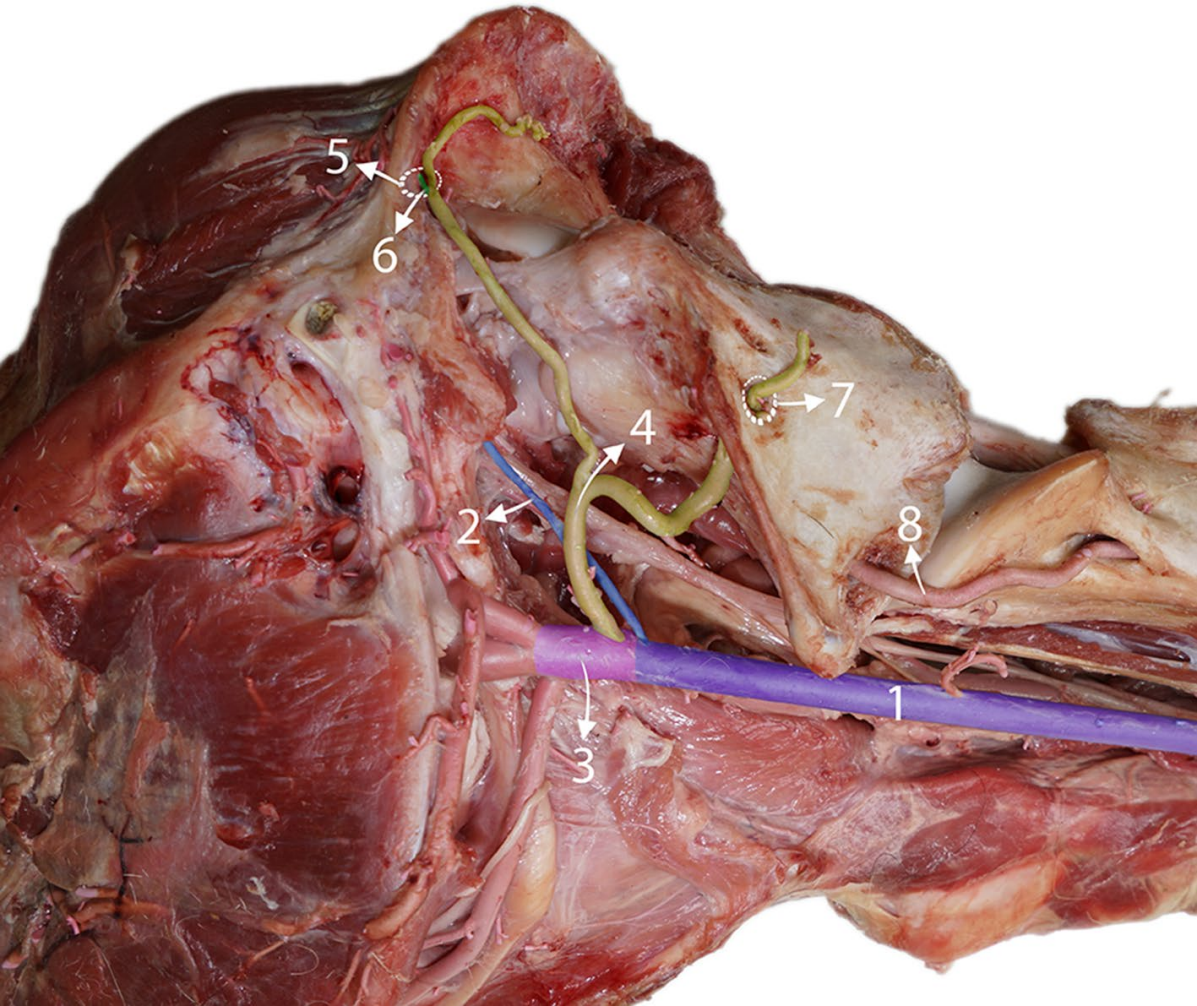
Lateral view of a dissected camel head and neck showing the rubber-casted branches of the common carotid artery, highlighted and colored to enhance visibility and understanding. 1, Common carotid artery; 2, internal carotid artery; 3, external carotid artery; 4, occipital artery; 5, mastoid foramen; 6, caudal meningeal artery; 7, alar foramen, and 8, vertebral artery.

**Figure 7:**
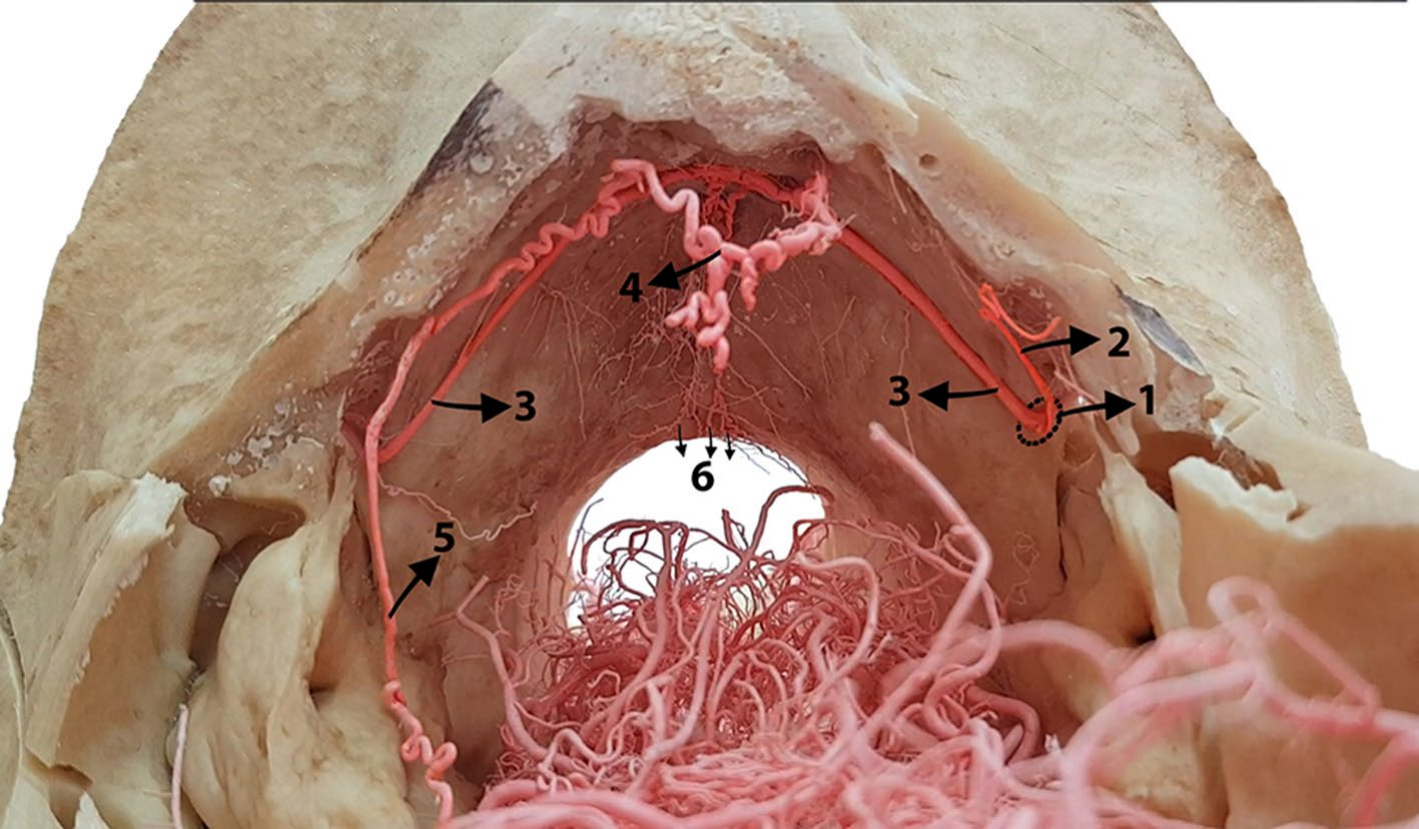
Rostral view of an exposed cranial cavity of a dromedary camel (brain removed), showing the branching patterns of the caudal meningeal arteries within the caudal cranial region. 1, Mastoid foramen; 2, first branch of caudal meningeal artery; 3, caudal meningeal artery; 4, rostral branches of caudal meningeal artery; 5, caudal branch of the right middle meningeal artery; 6, caudal radiating branches of caudal meningeal artery.

The caudal meningeal artery originates from the occipital arteries and enters the cranial cavity via the mastoid foramen (Figs. 6 and 7). The occipital artery is the first major branch of the external carotid arteries. The occipital artery rises, following the posterior curve of the temporal crest and the caudal aspect of the squamous portion of the occipital bone. Along the way, it provides a caudodorsal branch that proceeds toward the atlantic fossa. This branch subsequently enters the alar foramen, where it meets and forms an anastomosis with the vertebral artery (Fig. 6). Following this bifurcation, the occipital artery continues to ascend, giving rise to the caudal meningeal artery that enters the cranium through the mastoid foramen. Beyond this point, the occipital artery branches further, providing multiple muscular branches that supply the ligamentum nuchae and the overlying muscles in the occipital region. The caudal meningeal artery originated from the condylar artery in one of the studied samples.

After entering the mastoid foramen, the caudal meningeal artery sends a thick branch (Fig. 7) that supplies the meninges of the lateral margin of the cerebellum. The caudal meningeal artery initially follows a linear pattern and runs within the groove of the caudal meningeal artery. However, it adopts a more tortuous route as it traverses the caudal surface of the cerebellum. This course ends in an anastomosis with its counterpart on the opposite side at the level of the parieto-occipital suture (Figs. 2 and 7). The caudal meningeal arteries send out numerous branches at this level, forming a network of arteries that supply the meninges covering the cerebellum (Figs. 1 and 2). Subsequently, the caudal meningeal artery bifurcates and radiates rostrally and caudally (Fig. 7). These branches ensure comprehensive vascularization of the meninges covering the cerebellum. In parallel, the caudally oriented branches provide an internal supply to the occipital bone. Following anastomosis, the caudal meningeal arteries take a superomedial course, providing a vascular supply to the falx cerebelli and dura mater lining the dorsocaudal portion of the cranial cavity (Figs. 1 and 2).

In addition to the rostral, middle, and caudal meningeal branches, our observations revealed the presence of supplemental accessory branches arising directly from the RERM (Figs. 1 and 5). These branches supply the meninges and cover the ventral and lateral frontal lobes (Figs. 1 and 5).

## Discussion

To the best of our knowledge, this is the first study to comprehensively outline the complex topologies and origins of all meningeal arteries in dromedaries. Owing to the lack of thorough investigations in this field, the precise definition and identification of these structures were challenging. While some studies have touched upon the role of the middle meningeal arteries in the formation of the RERM, extensive exploration of this and other meningeal arteries has not been the subject of any prior research or descriptive studies in animals. The meningeal arteries comprise arterial branches that extend between the dura mater and the bone, supplying both structures. We found that the rostral meningeal artery is derived from the external ethmoidal artery as it passes through multiple ethmoid foramina. This finding is in contrast to those described by Kanan^19^, who reported that the rostral meningeal artery is a branch of the anterior cerebral artery that supplies the trigonum olfactorium and anterior part of the dura mater. Despite the lack of comprehensive investigations on the origin and course of the rostral meningeal arteries in animals, some studies have suggested that the rostral meningeal arteries may be involved in the development of the rete in goats^20^ and arterial circles in dogs^3^. In goats, three or four rostral meningeal arteries arise from the maxillary artery anterior to the ophthalmic artery and enter the cranium through the foramen orbito-rotundum^20^. According to Parkash and Jain^20^, a network of three to four rostral meningeal arteries, two to three middle meningeal arteries, the caudal meningeal artery, and the cerebrospinal artery join the RERM in goats. In dogs, the rostral meningeal artery travels dorsally through the dura at the caudal margin of the cribriform plate, moves through the inner layer of the frontal bone, and anastomoses rostrally with the middle meningeal artery to form an arterial circle on the lateral wall of the cribriform plate. In dogs, many smaller branches leave the arterial circle and reunite to create an ethmoidal rete^3^.

Among the three meningeal arteries, the middle meningeal artery has the most extensive supply region, providing blood to approximately two-thirds of the meninges in the brain^21^. Our findings in camels confirm that the supply of the middle meningeal artery is widespread. Zguigal^2^ reported that the middle meningeal artery shares a trunk with the rostral tympanic artery, arising from the inferior alveolar artery.

In contrast, Badawi et al.^22^ found that the maxillary artery of camels releases the alveolar, buccal, and middle meningeal arteries. In comparison, our study found that the middle meningeal artery arose from the RERM and, in one case, the rostral branch of the middle meningeal artery was derived from the rostral branches of the maxillary artery.

In cats and lions, the middle meningeal artery originates from the posteromedial wall of the maxillary artery, passes through the oval foramen to enter the cranial cavity, and runs posteromedial along the posterior margin of the rete^23,24^. In canines, the middle meningeal artery separates from the dorsal surface of the maxillary artery when entering the alar canal^3^. According to O'Brien et al.^25^, the middle meningeal artery in cattle and giraffes originates from the rostral auricular artery before entering the temporal meatus through the retro-articular foramen and supplies the caudolateral aspect of the meninges. According to studies by Zguigal^2^ and Kieltyka-Kurc et al.^5^, the middle meningeal artery enters the cranium through the oval foramen parallel to the mandibular nerve and then gives off numerous branches to supply the meninges in camels.

The middle meningeal arteries contribute to the caudal root of the RERM in camels^2,7^. The contribution of the middle meningeal artery to the RERM has been emphasized in animals such as giraffes, cattle, goats, dog, and cats^16–19,25^. Several studies have addressed the significance of the RERM, a network of anastomosing arteries found exclusively in artiodactyls and carnivores^2,26–30^. The RERM pools blood from different arteries and acts as a reservoir for the afferent blood. RERM is believed to reduce cerebral blood pressure from afferent arteries^7,31,32^. The RERM also maintains a lower brain blood temperature by dissipating heat from warm arterial blood^2,6,25,33,34^. Our findings suggest that meningeal arteries emerging from the RERM may have a significant thermoregulatory function in camels. The rostral ethmoidal rete mirabile is an important anatomical feature situated within the venous sinus inside the camel's cranial cavity. This sinus plays an essential role in receiving cooler arterial blood from veins in the regions surrounding the eyes, forehead, and nasal cavities. This arriving blood is notably cooler than the blood within the RERM itself. This unique setup results in the cooling of the arterial blood contained in the rete mirabile before it is directed to supply the brain. Our research lends strong support to the idea that this mechanism contributes to maintaining the brain at a lower temperature than the rest of the body^25,33,35,36^. This difference in temperature is enhanced by the cooling effect of the middle meningeal artery which originates from the RERM. This cooling process in the region supplied by the middle meningeal artery plays a crucial role in the overall thermal regulation of the camel's brain. This suggests that these arteries are part of the cerebral cooling system that is crucial for preserving brain function in extreme environments, and act as vital components in maintaining the brain's thermal balance. Evans and De Lahunta^3^ found that the middle meningeal artery in dogs exits the ramus anastomoticus and runs along the calvaria's vascular groove, splitting into rostral and caudal branches. Our study found that camels exhibited similar branching patterns, with the rostral branch of the middle meningeal artery supplying the meninges covering the parietal and temporal lobes, and the caudal branch of the artery supplying the meninges covering the occipital lobe across all examined specimens. Additionally, our study revealed that camels have accessory meningeal branches that emerge directly from the rete and primarily supply the meninges covering the ventral and lateral aspects of the frontal lobe.

The caudal meningeal arteries are branches of the occipital artery that enter the cranial cavity via the mastoid foramen. According to Zguigal^2^, the caudal meningeal artery is the terminal branch of the caudal auricular artery, originating from the external carotid artery. Tayeb^37^ reported that the caudal meningeal artery is the terminal branch of the auriculomeningeal artery and the only collateral branch of the external carotid artery. According to Benkoukous^38^, the external carotid artery gives rise to the common trunk of the caudal auricular and caudal meningeal arteries in camels. In our study, we observed that, except for one sample, the caudal meningeal artery consistently originated from the occipital artery and entered the cranial cavity through the mastoid foramen. In this exception, the caudal meningeal artery originated from the condylar artery, which is usually a branch of the occipital artery in the camel. This is similar to the observation made by O’Brien et al.^25^ in giraffes, where the large caudal meningeal artery arises from the occipital artery and enters the skull via the mastoid foramen, and a small caudal meningeal artery departs rostrally from the condylar artery. The caudal meningeal arteries in dogs branch off the occipital branch along the nuchal crest of the occipital bone, branching into the dura of the dorsocaudal cranial cavity through the mastoid foramen and supplying some branches to the tentorium cerebelli just dorsal to the petrosal part of the temporal bone^3^. In horses, the occipital artery supplies blood to the nuchal region, caudal meninges, middle and inner ears^39^.

In conclusion, this study is the first to comprehensively describe the origins and courses of all meningeal arteries in dromedaries, filling a significant gap in the existing animal literature. Notably, the referenced information on the meningeal arteries was not the focus of the cited studies. This indicates a lack of dedicated research on this specific topic and highlights the novelty of our research in filling this substantial gap. This study identified often overlooked arterial sources in the meninges, such as the additional accessory branches emerging directly from the RERM and supplying most of the meninges of the frontal lobes. We were able to account for variations owing to the substantial sample size. The origin and course of camel meningeal arteries suggest their essential role in efficient brain cooling mechanisms, explaining their ability to survive in extremely harsh environments. Understanding the detailed anatomy of meningeal artery branching patterns in dromedaries is essential for both veterinary practitioners and researchers. This information can help in the diagnosis and treatment of various neurological conditions in dromedaries, ensuring the optimal health and well-being of these animals. Further research in this field can contribute to advancements in veterinary medicine and enhance our understanding of the intricate circulatory system in dromedaries, emphasizing the uniqueness of their anatomical structure.

## Methods

This study was conducted in accordance with the guidelines of the Animal Research Ethics Committee of United Arab Emirates University. In this study, we injected various casting materials into the right and left common carotid arteries of 20 freshly slaughtered male Omani dromedaries (2–6 years old) obtained from Al Ain City Municipality Camel Slaughterhouses, as described in our previous studies^5^.

We injected liquid polyurethane resin (Polytek EasyFlo 60) into the common carotid arteries of 10 dromedary heads, and red latex neoprene (Globalsil 20; Global Chemica) into the remaining 10 heads. Owing to the flexibility of latex, specimens injected with latex were dissected to remove the camel brains, including their meninges, from the cranial cavity while maintaining intact arteries. We used the resin-injected specimens to create three-dimensional models of the meningeal arteries within the cranial cavity after removing the brain using a high-pressure washer. This method was used to remove brain tissue without damaging the resin-cast fillings. High-resolution photographs of all the dissected specimens were captured using a Sony camera with a resolution of 42 megapixels.

## Data Availability

The datasets for this study can be made available by the corresponding author, without undue reservation.

## References

[1] Smuts MMS, Bezuidenhout AJ (1987). Anatomy of the Dromedary.

[2] Zguigal, H. Angioarchitecture of the nasal cavity and the carotid rete-cavernous sinus complex and their functional significance in the camel (*Camelus Dromedarius*) [Doctor of Philosophy]. Ames, Iowa: Iowa State University (1988).

[3] Evans HE, De Lahunta A (2012). Miller's anatomy of the dog-E-Book.

[4] Ocal MK, Erden H, Ogut I, Kara MEA (1999). quantitative study of the circulus arteriosus cerebri of the camel (*Camelus dromedarius*). Anat. Histol. Embryol..

[5] Kiełtyka-Kurc A, Frackowiak H, Zdun M, Nabzdyk M, Kowalczyk K, Tokacz M (2014). The arteries on the base of the brain in the camelids (Camelidae). Ital. J. Zool..

[6] Jerbi H, Khaldi S, Pérez W (2016). Morphometric study of the rostral epidural rete mirabile in the dromedary (*Camelus dromedarius*, Linnaeus 1758). Int. J. Morphol..

[7] Al-Aiyan A (2019). Descriptive analysis of cerebral arterial vascular architecture in dromedary camel (*Camelus dromedarius*). Front. Neuroanat..

[8] Al Aiyan A, Balan R (2023). Mapping the branching pattern of the middle cerebral artery in the camel (*Camelus dromedarius*): A comprehensive anatomical analysis. Front. Vet. Sci..

[9] Chmielewski P, Skrzat J, Walocha J (2013). Clinical importance of the middle meningeal artery. Folia Med. Cracov..

[10] Kresimir-Lukic I, Gluncic V, Marusic A (2001). Extracranial branches of the middle meningeal artery. Clin. Anat..

[11] Kornieieva M, Hadidy A, Zhuravlova I (2015). Variability of the middle meningeal artery subject to the shape of skull. J. Neurol. Surg. B Skull Base..

[12] Paiva WS (2014). Computed tomography angiography for detection of middle meningeal artery lesions associated with acute epidural hematomas. BioMed. Res. Int..

[13] Yu J, Guo Y, Xu B, Xu K (2016). Clinical importance of the middle meningeal artery: A review of the literature. Int. J. Med. Sci..

[14] Sakata H (2009). Serial angiography of dynamic changes of traumatic middle meningeal arteriovenous fistula, case report. Neurol. Medico-chirurg..

[15] Nieuwenhuys R, Donkelaar HJ, Nicholson C (1998). The Central Nervous System of Vertebrates.

[16] Daniel PM, Dawes JDK, Prichard MML (1953). Studies of the carotid rete and its associated arteries. Philos. Trans R. Soc. Lond. B Biol. Sci..

[17] Simoens P, Lauwers H, Geest JP, Schaepdrijver L (1987). Functional morphology of the cranial Retia mirabilia in the domestic mammals. Schweiz. Arch. Tierheilkd..

[18] Kapoor K, Kak VK, Singh B (2003). Morphology and comparative anatomy of circulus arteriosus cerebri in mammals. Anat. Histol. Embryol..

[19] Kanan CV (1970). The cerebral arteries of camelus dromedaries. Acta anatomica: International journal of anatomy, embryology. Cell Biol..

[20] Parkash T, Jain RK (2014). Blood supply to the brain in goat (*Capra hircus*). Indian J. Vet. Anat..

[21] Martins C (2005). Microsurgical anatomy of the dural arteries. Oper. Neurosurg..

[22] Badawi H, El-Shaib M, Kenawy A (1977). The arteria maxillaris of the camel (*Camelus dromedarius*). Anat. Histol. Embryol..

[23] Takemura A (1982). The rete mirabile of the maxillary artery in the cat. Okajim. Folia Anatom. Jap..

[24] Hseih HM, Takemura A (1994). The rete mirabile of the maxillary artery in the lion (*Panthera leo*). Okajim. Folia Anatom.Jap..

[25] O’Brien HD, Gignac PM, Hieronymus TL, Witmer LM (2016). A comparison of postnatal arterial patterns in a growth series of giraffe (Artiodactyla: *Giraffa camelopardalis*). Peer J..

[26] Lesbre, F. X. Recherches anatomiques sur les Camélidés. De Lyon. (Archives du Muséum d’Histoire Naturelle de Lyon; vol 8). https://www.persee.fr/issue/mhnly_0374-5465_1903_num_8_1 (1903).

[27] García-Villalón AL (1989). Mechanics of arteries forming the carotid rete of goat and cattle. Microvasc. Res..

[28] Ocal MK, Erden H, Ogut I, Kara ME (1998). A quantitative study on the retial arteries in one-humped camels. Ann. Anat..

[29] Zdun M (2013). The arteries of brain base in species of Bovini tribe. Anat. Rec..

[30] Sorby-Adams AJ, Vink R, Turner RJ (2018). Large animal models of stroke and traumatic brain injury as translational tools. Am. J. Physiol. Regul. Integr. Comp. Physiol..

[31] Kiełtyka-Kurc A, Frackowiak H, Brudnicki W (2015). The arteries of brain base in species of the cervid family. Anat. Rec..

[32] Al Aiyan A (2021). Vertebrobasilar contribution to cerebral arterial system of dromedary camels (*Camelus dromedarius*). Front. Vet. Sci..

[33] Samara E, Ayadi M, Aljumaah A (2013). Thermophysiological study in lactating and dry camels (*Camelus dromedarius*) under summer conditions. Emir. J. Food Agric..

[34] Deepthi S, Suseelamma D, Pramod-Kumar D, Saradadevi SS, Subhadradevi V (2016). Comparative study of formation of Circle of Willis in human and sheep brain. J. Anat. Soc. India.

[35] Hayward JN, Baker MA (1969). A comparative study of the role of the cerebral arterial blood in the regulation of brain temperature in five mammals. Brain Res..

[36] Taylor CR, Lyman CP (1972). Heat storage in running antelopes: Independence of brain and body temperatures. Am. J. Physiol..

[37] Tayeb MAF (1951). Study on the blood supply of the camel’s head. Br. Vet. J..

[38] Benkoukous, M. Irrigation aretrielle de la tete chez le dromadaire (*Camelus dromedarius*). These de Doctorat Veterinaire. I.A.V. Hassan II. Rabat-Maroc (1983).

[39] Konig, H. E., Liebich, H. G. & Cerveny, C. *Veterinary Anatomy of Domestic mammals Text Book and Color Atlas*, 4 Ed 489–518 (Germany, 2009).

